# Inversion of winter wheat leaf area index from UAV multispectral images: classical vs. deep learning approaches

**DOI:** 10.3389/fpls.2024.1367828

**Published:** 2024-03-14

**Authors:** Jiaxing Zu, Hailong Yang, Jiali Wang, Wenhua Cai, Yuanzheng Yang

**Affiliations:** ^1^ Key Laboratory of Environment Change and Resources Use in Beibu Gulf, Ministry of Education, Nanning Normal University, Nanning, China; ^2^ Guangxi Key Laboratory of Earth Surface Processes and Intelligent Simulation, Nanning Normal University, Nanning, China; ^3^ School of Geography and Planning, Nanning Normal University, Nanning, China

**Keywords:** leaf area index, multispectral, UAV, machine learning, convolutional neural network, orthophoto

## Abstract

Precise and timely leaf area index (LAI) estimation for winter wheat is crucial for precision agriculture. The emergence of high-resolution unmanned aerial vehicle (UAV) data and machine learning techniques offers a revolutionary approach for fine-scale estimation of wheat LAI at the low cost. While machine learning has proven valuable for LAI estimation, there are still model limitations and variations that impede accurate and efficient LAI inversion. This study explores the potential of classical machine learning models and deep learning model for estimating winter wheat LAI using multispectral images acquired by drones. Initially, the texture features and vegetation indices served as inputs for the partial least squares regression (PLSR) model and random forest (RF) model. Then, the ground-measured LAI data were combined to invert winter wheat LAI. In contrast, this study also employed a convolutional neural network (CNN) model that solely utilizes the cropped original image for LAI estimation. The results show that vegetation indices outperform the texture features in terms of correlation analysis with LAI and estimation accuracy. However, the highest accuracy is achieved by combining both vegetation indices and texture features to invert LAI in both conventional machine learning methods. Among the three models, the CNN approach yielded the highest LAI estimation accuracy (*R* ^2^ = 0.83), followed by the RF model (*R* ^2^ = 0.82), with the PLSR model exhibited the lowest accuracy (*R* ^2^ = 0.78). The spatial distribution and values of the estimated results for the RF and CNN models are similar, whereas the PLSR model differs significantly from the first two models. This study achieves rapid and accurate winter wheat LAI estimation using classical machine learning and deep learning methods. The findings can serve as a reference for real-time wheat growth monitoring and field management practices.

## 1 Introduction

Wheat, a globally cultivated food grain crop, plays a pivotal role in sustaining approximately 35% of the world’s population. Its growth and yield are paramount for safeguarding food security (IDRC, 2010). Leaf area index (LAI) is intricately linked to fundamental biophysical processes like vegetation light absorption, evapotranspiration, and productivity (Du et al., 2022; Ma et al., 2023). Therefore, obtaining real-time and reliable LAI estimations is essential for assessing wheat growth potential and providing timely technical guidance for subsequent field management practices (Bellis et al., 2022; Yang et al., 2019).

Currently, two widely used methods for LAI measurement are direct and indirect. While direct measurement through field observations boasts high accuracy, it is labor-intensive and destructive, making it impractical for large scale assessments (Castro-Valdecantos et al., 2022). Alternatively, indirect methods are based on the Beer-Lambert Law theory and typically involve optical instruments for bottom-up hemispherical photography and spectral reflectance measurements (Apolo-Apolo et al., 2020; Yan et al., 2016). However, both direct and indirect approaches are inadequate for extending LAI estimation to larger scales or regions. Remote sensing technology offers a viable solution for this challenge. In recent decades, researchers have successfully utilized medium- and low-resolution satellite datasets, such as Landsat, MODIS (Moderate Resolution Imaging Spectroradiometer), and AVHRR (Advanced Very High-Resolution Radiometer), to retrieve broad-scale LAI (Xiao et al., 2016; Kang et al., 2021). Yet, LAI estimation based on satellite data falls short in providing refined field-scale monitoring. Unmanned aerial vehicles (UAVs) offer substantial advantages over satellites in terms of enhanced temporal and spatial resolution, alongside greater flexibility (Li et al., 2019; Yin et al., 2023). Multispectral cameras are a popular choice among UAV sensors, it can capture spectral information in the red-edge and near-infrared bands, which are crucial for analyzing vegetation (Yao et al., 2019). These spectral bands and the vegetation indices have been effectively employed to predict crop LAI, demonstrating commendable accuracy (Liu et al., 2021; Wang et al., 2020). However, limitations arise when estimating LAI solely using visible or multispectral vegetation indices, especially in the presence of high crop cover and saturation phenomena (Corti et al., 2022; Zhou et al., 2017). To address these limitations, some researchers have incorporated texture features alongside vegetation indices for crop parameter estimation (Liu et al., 2023b; Xu et al., 2022). For example, Liu, et al. (Liu et al., 2023a) investigated the use of vegetation indices, structural information, and texture features for estimating the leaf area index (LAI) of winter wheat. The model utilizing only texture features achieved the highest accuracy of 0.56 (*R* ^2^), surpassing models based solely on vegetation indices or structural information. Texture’s success in LAI estimation stems largely from its ability to capture spatial information about crops (Zhang et al., 2022a; Liu et al., 2018). However, the existing research methods cannot accurately reflect the complex changes in the canopy structure at different growth stages. It is necessary to explore the extent to which vegetation index and texture features can improve the estimation of wheat LAI, especially in the canopy closure stage and heading stage.

The inversion model plays a pivotal role in accurately quantifying the crop LAI. The traditional estimation methods primarily rely on the empirical relationship between spectral features and crop parameters, often employing linear regression. While this method is straightforward to compute and implement, it is prone to regional variations and discrepancies across different crop types, even with similar predictors (Chen et al., 2002; Zhou et al., 2021). Additionally, traditional statistical regression models face limitations in handling complex nonlinear and multicollinearity relationships between LAI and spectral features. In recent years, numerous machines learning algorithms, including partial least squares regression (PLSR), random forest (RF), and decision trees (DT) have gained widespread adoption in remote sensing parameter inversion. These algorithms excel in modeling nonlinearity and heteroscedasticity relationships among various feature types (Jordan and Mitchell, 2015; Lary et al., 2016). However, the process of deriving and selecting a large number of characteristic bands can be impractical and inefficient, making it challenging to identify the most effective set of predictors and potentially leading to suboptimal performance. The emergence of deep learning (DL) techniques has revolutionized agricultural applications, offering robust and intelligent solutions (Osco et al., 2021). Unlike the shallow neural network approach, deep learning is characterized by a significantly increased number of successively connected neural layers, allowing them to extract more complex relationships (Kattenborn et al., 2021). Convolutional neural networks (CNN) are particularly adept at analyzing spatial patterns. Designed for spatial feature extraction, the CNN model possesses the advantage of directly processing raw images, eliminating the need for extensive pre-processing (Liang et al., 2018). The efficacy of UAV-based multispectral data in estimating crop leaf area index (LAI) has fueled advances in agricultural remote sensing (Guo et al., 2023; Wittstruck et al., 2022). Despite the widespread utilization of algorithms for predicting crop LAI, there remains a paucity of studies comparing the performance of wheat LAI estimation using both classical machine learning and deep learning approaches based on multispectral UAV data.

This study leverages UAV-derived multispectral data to address two key objectives: 1) to compare the predictive potential of vegetation indices and textural features for estimating winter wheat LAI, 2) to evaluate the performance of traditional machine methods (PLSR and RF) and deep learning method (CNN) for predicating wheat LAI. We hope our findings will provide technical basis and references for estimating key crop parameters during critical growth stages of wheat.

## 2 Materials and methods

### 2.1 Study area

**Figure 1:**
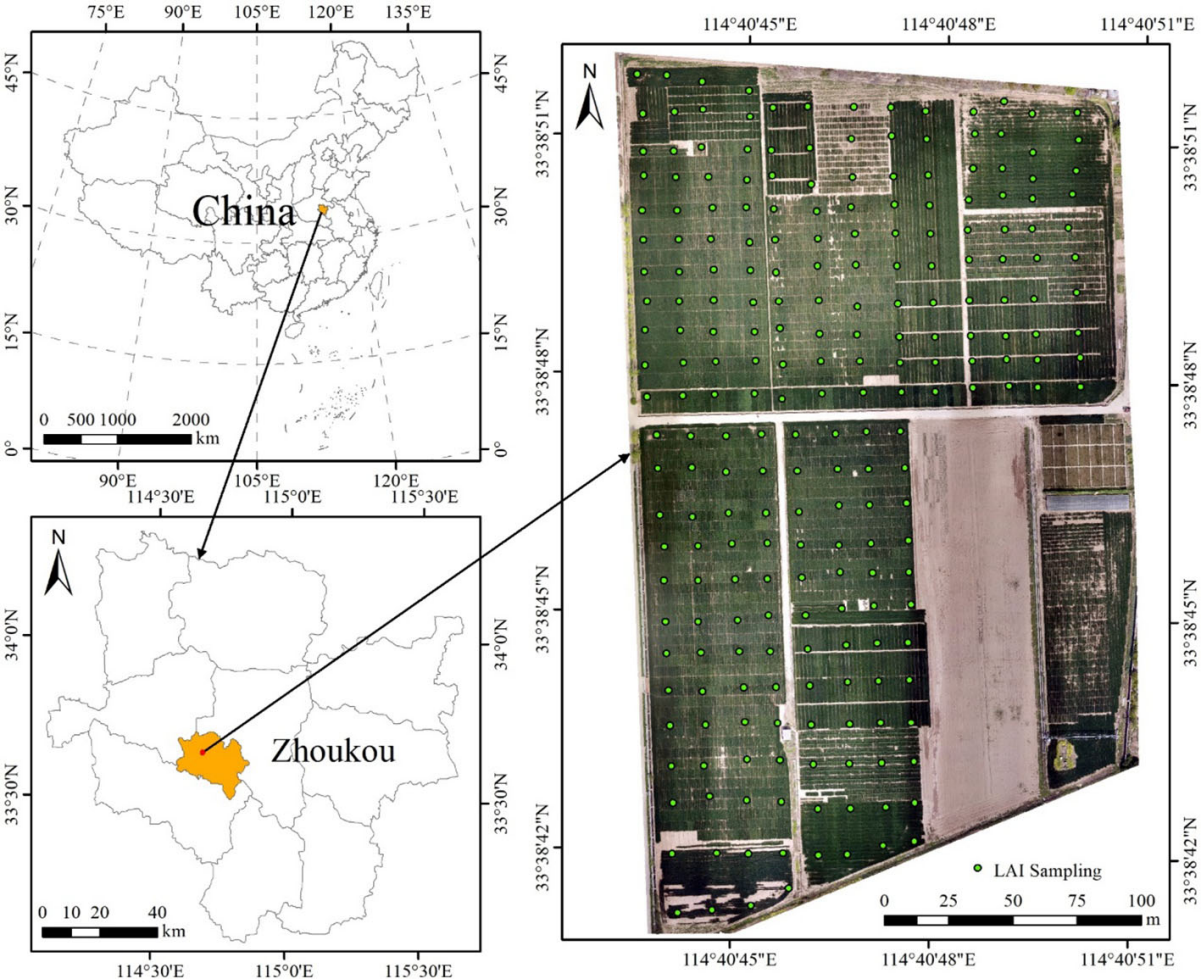
Location of the study area and LAI sampling points distribution.

The winter wheat study area is located in the winter wheat breeding experimental field of Zhoukou Academy of Agricultural Sciences, Zhoukou, Henan province, China (114°41 ‘E, 33°39 ‘N), as shown in **Figure 1**. The primary crops cultivated are winter wheat and summer corn. The study area cultivates dozens of different wheat varieties with the purpose of displaying wheat varieties and conducting breeding screenings, which enhances the variability of the image dataset. Notably, the winter wheat was sown in October 2022, followed the same management practices as the local winter wheat crop.

### 2.2 Unmanned aerial vehicle data collection

On April 5, 2023, the critical period of wheat growth, specifically the heading stage, was captured using UAV remote sensing imagery. For this observation, a DJI Phantom 4 multispectral UAV (DJI Technology, Ltd., Shenzhen, China) was utilized to acquire images of the wheat canopy. The UAV’s RGB sensor provided high resolution visible light imagery, while its five monochrome sensors captured data across specific spectral bands (details in **Table 1**). The UAV has an integrated light intensity sensor on top, which can obtain solar irradiance information to compensate for illumination of the image and exclude interference from ambient light. In addition, the UAV platform is equipped with a real-time kinematic (RTK) module, which enables centimeter-level localization accuracy. For mission planning, DJI GS Pro software is employed to plan the route. Data collection was conducted at an altitude of 70 m under clear and windless sky conditions between 10:00 and 14:00. The spatial resolution of the captured images is 3.7 cm, and the drone’s flight speed is 5 m/s. The mission included an 80% heading overlap, a 70% side overlap, resulting in the acquisition of 2382 images. Using DJI Terra software, the acquired raw images were stitched together following below steps: (1) import all the images to the software; (2) perform aerial triangulation processing to calculate the sensor’s position and orientation during imaging, as well as generating a spare point cloud of the captured objects; (3) validate the quality, proceed to multispectral stitching reconstruction, and finally produce the ortho-mosaic images covering the study area.

**Table 1:** The vegetation indices adopted in this study.

Spectral Indices	Formula	References
Normalized difference vegetation index (NDVI)	(NIR-R)/(NIR+R)	(Tao et al., 2020b)
Excess blue index (ExB)	1.4b-g	(Mao et al., 2003)
Excess green index (ExG)	2g-r-b	(Woebbecke et al., 1995)
Excess red index (ExR)	1.4r-g	(Meyer and Neto, 2008)
Excess green minus excess red index (ExGR)	(2g-r-b)-(1.4r-g)	(Niu et al., 2018)
Modified excess green index (MExG)	1.262g-0.884r-0.311b	(Sun et al., 2016)
Visible-band difference vegetation index (VDVI)	(2g-r-b)/(2g+r+b)	(Wang et al., 2015)
Color index of vegetation extraction (CIVE)	0.441r-0.811g+0.385b+18.78745	(Kataoka et al., 2003)
Normalized green-red difference index (NGRDI)	(g-r)/(g+r)	(Hunt et al., 2005)
Blue-green ratio index (BGRI)	b/g	(Sellaro et al., 2010)
Red green ratio index (RGRI)	r/g	(Verrelst et al., 2008)
Vegetation index (VEG)	g/(r^0.667^b^0.333^)	(Hague et al., 2006)
Combined indices 1 (COM1)	ExG+CIVE+ExGR+VEG	(Guijarro et al., 2011)
Combined indices 2 (COM2)	0.36ExG+0.47CIVE+0.17VEG	(Guerrero et al., 2012)
Ratio vegetation index (RVI)	NIR/R	(Yu et al., 2020)
Different vegetation index (DVI)	NIR-R	(Jordan, 1969)
Green normalized difference vegetation index (GNDVI)	(NIR-G)/(NIR+G)	(Gitelson et al., 1996)
Blue normalized difference vegetation index (BNDVI)	(NIR-B)/(NIR+B)	(Yang et al., 2004)
Renormalized difference vegetation index (RDVI)	(NIR-R)/(NIR+R)^0.5^	(Zheng et al., 2020)
Normalized difference red edge index (NDRE)	(NIR-RE)/(NIR+RE)	(Fitzgerald et al., 2010)
Enhanced vegetation index (EVI)	2.5(NIR-R)/(NIR+6R-7.5B+1)	(Peng et al., 2021)
Leaf chlorophyll index (LCI)	(NIR-RE)/(NIR+R)	(Datt, 1999)
Optimized soil adjusted vegetation index (OSAVI)	(NIR-Red)/(NIR+Red+0.16)	(Hunt, 2011)

In the table above, R, G, B, RE and NIR indicate the spectral reflectivity in the red (450 nm), green (560 nm), blue (650 nm), red-edge (730 nm) and near-infrared (840 nm) bands, respectively. r= R/(R+G+B), g=G/(R+G+B), b=B/(R+G+B).

### 2.3 Field LAI data collection

Ground-based measurements of wheat LAI were conducted within three days of acquiring UAV images. The LAI-2200C canopy analyzer (LI-COR Biosciences, Inc., Lincoln, NE, USA) was utilized to obtain non-destructive information on the leaf area index. LAI measurements were conducted under controlled weather conditions, ensuring cloudy or clear, cloud-free weather with uniform cloud thickness. A total of 234 wheat quadrats (1×1m), evenly distributed across the study area, were selected for measurement (**Figure 1**), and the wheat growth within these quadrats was uniform. During measurements, the radiative value (A) was initially obtained above the wheat canopy, followed by the acquisition of four radiative values (B) below the diagonal wheat canopy in the two ridges. After the measurement, the instrument automatically computes the LAI values of the sampled points. Four sets of LAI values were measured within each sample square. During measurements, a 45° view cap was utilized to prevent the entry of the observer or direct light into the sensor’s field of view. Additionally, four A values were measured for scattering correction in the presence of sunlight exposure, aiming to mitigate errors caused by sunlight scattering. The centimeter-level differential localization service is provided by Qianxun SI (Shanghai, China), the sample number and its coordinate information were recorded by handheld RTK equipment (Qianxun SI, Ltd., Shanghai, China) at the center of each sample.

### 2.4 Data preparation

#### 2.4.1 Extraction of vegetation index and texture feature

Vegetation indices provide a simple and effective means to reflect vegetation growth characteristics and are extensively employed for monitoring physiological and biochemical parameters, including crop LAI (Bendig et al., 2015; Tao et al., 2020b). For this study, 23 vegetation indices demonstrating a strong correlation with LAI, including NDVI, NDRE, and OSAVI, were chosen for estimation and modeling, building on previous research findings (**Table 1**). The mean value of the vegetation index computed for all pixels in each wheat quadrant corresponds to the measured LAI value on the ground, and the LAI is then estimated. ​The vegetation index is calculated and the spectral average is obtained in R ver. 4.3.1.

Texture features were extracted using the gray level co-occurrence matrix (GLCM), a powerful spatial analysis technique that captures the relationship between pixels, and has demonstrated effectiveness in extracting crop-related information in numerous studies (Ilniyaz et al., 2023). In this study, ENVI software was employed to extract 8 GLCM texture features from each of the 5 multispectral bands, including dissimilarity (dis), variance (var), entropy (ent), mean (mean), synergism (hom), second moment (sec), correlation (cor) and contrast (con). During extraction, 3×3 windows were employed, and calculations were performed at a 45° angle. Previous studies have shown that the constructed GLCM texture features are essentially independent of different directions and window sizes concerning the correlation with vegetation physiological parameters (Liu et al., 2022a; Fu et al., 2020). In order to reduce data dimensionality, this study extracted features using a 3×3 window and calculated them at a 45° angle. This process resulted in the extraction of a total of 40 texture features. To enhance clarity, the band name and texture features are linked with “_”. For instance, red_mean and red_var indicate the extraction of mean (mean) and variance (var) texture features from the red band, respectively.

#### 2.4.2 Feature optimization

This study utilizes the random forest algorithm to compute the weights assigned to each feature. Additionally, Pearson correlation analysis quantifies the linear relationship between each feature and LAI. The optimal feature selection is performed by synthesizing the correlation coefficients between each image feature and LAI, as well as the weights of each feature. Image features with weights exceeding 0.03 and displaying extremely significant correlation with LAI (without restricting the size of correlation coefficients) were chosen for subsequent LAI estimation of winter wheat. Following optimization, a total of 9 vegetation indices and 10 texture features were identified (**Table 2**).

**Table 2:** Feature optimization result.

Feature type	Feature name
Vegetation index	BNDVI、DVI、GNDVI、LCI、NDRE、NDVI、OSAVI、RDVI、RVI
Texture feature	red_mean、red_var、red_hom、red_con、red_dis、red_ent、red_sec、red_cor、nir_mean、nir_cor

### 2.5 Model construction

#### 2.5.1 Feature-based machine learning model construction

To compare performance with deep learning methods, this study employed two classical machine learning models: partial least squares regression (PLSR) and Random Forest (RF). These models are extensively used in remote sensing and LAI inversion. PLSR is a multivariate regression analysis that combines advantages of traditional methods like principal component analysis and multivariate linear regression. PLSR adopts the method of data dimensionality reduction and increases the covariance between independent variables and dependent variables through the sequential selection of orthogonal factors, which reduces redundancy and improves the computational efficiency of the mode (Shao et al., 2023). In cases of datasets with numerous variables and multiple correlations between independent and dependent variables, the traditional linear regression method lacks precision in analyzing dependent variables, leading to low model accuracy. In contrast, the PLSR method enhances model accuracy under such circumstances. Furthermore, the resulting regression model will encompass all the information from independent variables and furnish a comprehensive explanation of the model’s regression coefficients (Tang et al., 2022; Tao et al., 2020a). The optimal number of explanatory variables for PLSR in this study was determined based on the root-mean-square error of LAI inversion accuracy (Cui and Kerekes, 2018), with default values adopted for other parameters. RF is an integrated learning algorithm founded on multiple decision trees and bagging technology introduced by Breiman (2001) in 2001. The training set is formed by putting samples back and taking samples multiple times, and then the prediction results are averaged through a combination of decision trees. The RF performance hinges on two crucial parameters: the number of decision trees and the number of split nodes. Generally, as the number of decision trees increases, the prediction error of the model gradually decreases and stabilizes. Following parameter tuning, the number of decision trees in this study is set to 1000, and the number of split nodes adopts the default value (1/3 of the number of variables). In R, the “pls” and “randomForest” packages were used to construct the model and adjust the parameters. The independent variable of the input model was the mean vegetation index and the mean texture features in the 1×1m quadrat, and the dependent variable was the ground measured value of LAI. The selection of training and test samples is consistent with CNN and will be described in the following section.

#### 2.5.2 Image based CNN model construction

Given the augmented number of feature variables, expressing them through simple linear relations becomes challenging. Therefore, the model requires enhanced capability for nonlinear fitting. CNN has gained widespread adoption due to its robust nonlinear fitting capability, yielding favorable outcomes (Luo et al., 2023). Consequently, this study also constructs a CNN model for mapping LAI. Due to the apparent difference in the range of values between the different bands, the direct input of the CNN may trigger large gradient updates and thus prevent the network from converging. To remove the effect of such differences, the standard normalization method was used in this study, where the mean value of each band image was subtracted to centralize and then divided by its standard deviation to finally obtain a mean value of 0.

**Figure 2:**
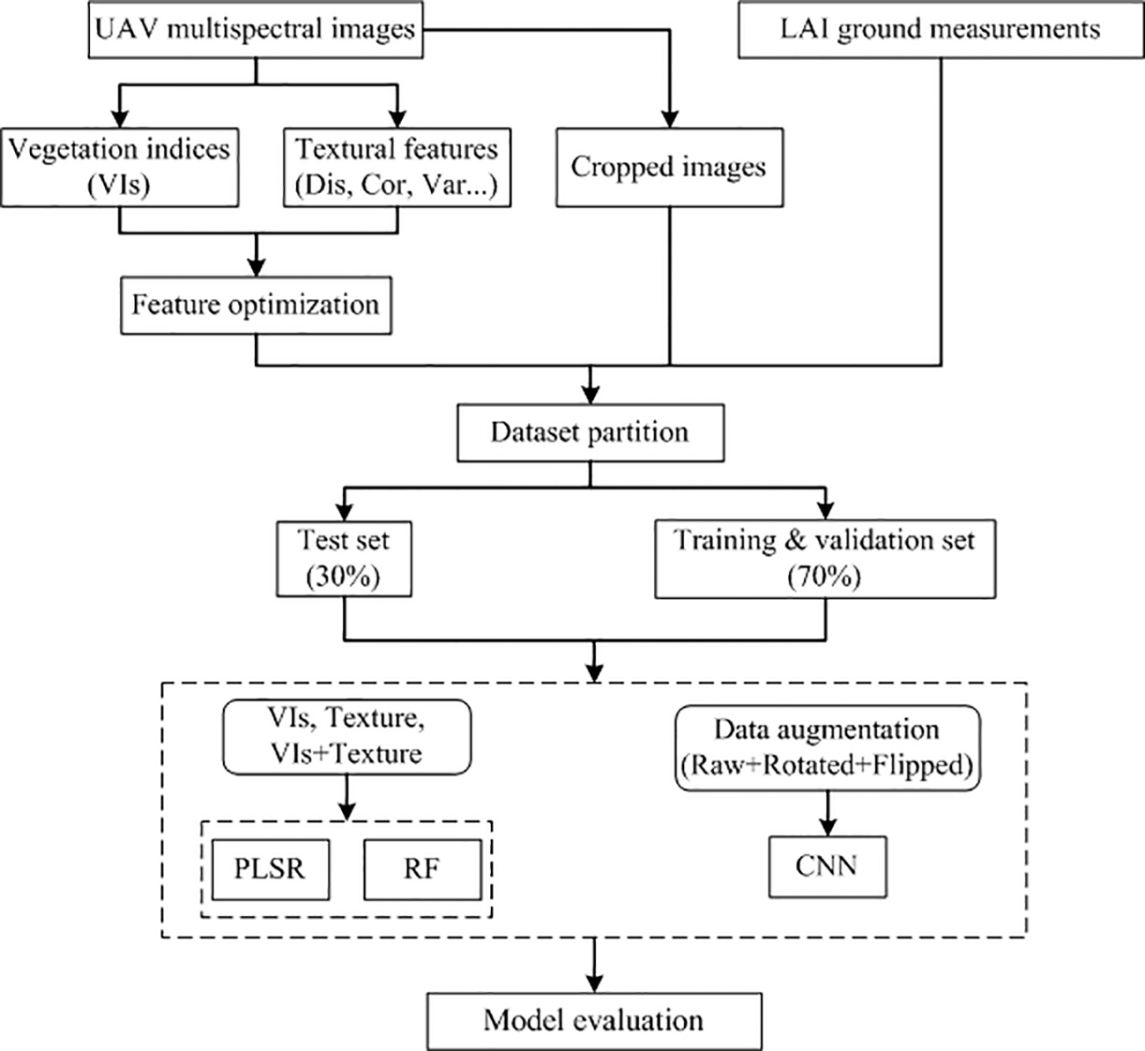
Flowchart of LAI estimation strategies.

The CNN model used in this study consists of convolutional layers, batch normalization layers, pooling layers, dropout layers, fully connected layers, and regression layers. The model employs the linear rectification function (ReLU) as the activation function, and the loss is calculated using the mean square error (MSE) function. The model is optimized using gradient descent (with momentum) optimizer, with a learning rate of 0.001. To mitigate overfitting, a dropout layer is incorporated, randomly excluding learned parameters from neurons with a probability 0.2. This reduces the network’s sensitivity to specific neuron weights, thereby improving the model’s generalization ability. The specific structure of CNN model is shown in **Table 3**. The input CNN requires 234 cropped quadrate images, with each containing 32 by 32 pixels. This study employs data augmentation to expand the sample dataset. Prior to expansion, 70% of the sample data (164 frames) is allocated for training and validation, while the remaining 30% (70 frames) is reserved for the test dataset. Rotation and flipping are widely used in data expansion (Xiong et al., 2017; Ma et al., 2019). In this study, these methods are applied to augment the training and validation datasets, while the test dataset remains unchanged for evaluation. Specifically, the images underwent rotations (90°, 180° and 270°) and flipping (horizontal, vertical), resulting in the creation of five extended datasets. Consequently, the final training and validation dataset was expanded sixfold, totaling 984 frames. Among these, 80% (787 frames) were allocated for training, and the remaining 20% (197 frames) for validation. The **Figure 2** shows the data augmentation traffic. The CNN model was implemented in R (4.3.1) using the “keras” and “tensorflow” packages. The computational setup includes an NVIDIA GeForce RTX 3090 graphics card (16GB video memory) (NVIDIA, Inc., Santa Clara, CA, USA), 64 GB RAM, and an Intel Core i7-11700 CPU (2.50 GHz, 16 cores) (Intel, Inc., Santa Clara, CA, USA).

**Table 3:** Structure of CNN.

Layer name	Filters	Kernel size	Output tensor
Convolutional layer 1	16	3×3	16×32×32
Batch normalization 1			16×32×32
Pooling layer 1			16×16×16
Convolutional layer 2	32	3×3	32×16×16
Batch normalization 2			32×16×16
Pooling layer 2			32×8×8
Convolutional layer 3	64	3×3	64×8×8
Batch normalization 3			64×8×8
Pooling layer 3			64×4×4
Convolutional layer 4	128	3×3	128×4×4
Batch normalization 4			128×4×4
Pooling layer 4			128×2×2
Dropout 1			128×2×2
Flatten layer 1			512
Dense layer 1			64
Dense layer 2			1

### 2.6 Model accuracy evaluation

This study employed the coefficient of determination (*R* ^2^), root mean square error (RMSE), and mean absolute error (MAE) as evaluation indices for assessing model accuracy. The *R* ^2^ signifies the agreement between estimated and measured values, with a value closer to 1 indicating a better fit of the model. The RMSE indicates the extent of deviation between estimated and measured values, with smaller values suggesting a better model fit. The MAE assesses the actual deviation between estimated and measured values, with smaller values indicating higher model accuracy. Both MAE and RMSE capture the average difference between estimated and measured values, with RMSE being more sensitive to large prediction errors. The formulas ((1), (2) and (3)) for these three indicators are as follows:


(1) $$R^{2}=\frac{\sum_{i=1}^{n}{\left(y_{i}-{\hat{y}}_{i}\right)}^{2}}{\sum_{i=1}^{n}{\left(y_{i}-\bar{y}\right)}^{2}}$$

(2) $$RMSE=\sqrt{\frac{\sum_{i=1}^{n}{\left(y_{i}-{\hat{y}}_{i}\right)}^{2}}{n}}$$

(3) $$MAE=\frac{1}{n}\sum_{i=1}^{n}\left|y_{i}-{\hat{y}}_{i}\right|$$

Where, $y_{i}$ and ${\hat{y}}_{i}$ represent the measured and estimated value of the i th sample, $\bar{y}\;$ is the average of all measured values, and n is the number of samples.

## 3 Results

### 3.1 Correlation analysis of vegetation index and texture features

**Figure 3:**
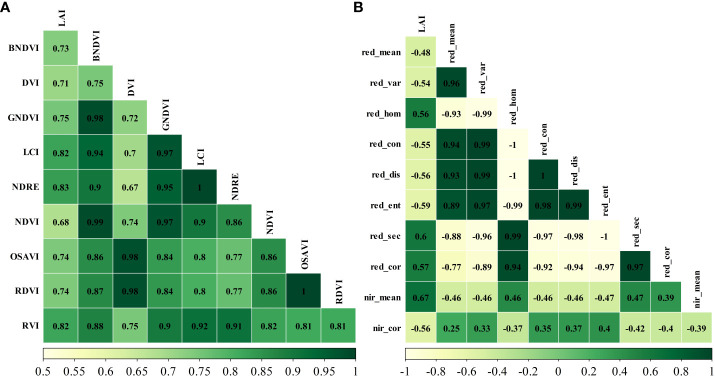
Correlation analysis results of vegetation index **(A)** and texture feature **(B)**. All correlation coefficients in the figure above pass the significance test at the p< 0.001 level.

Pearson correlation analysis was performed between texture features and vegetation indices after feature selection and LAI for winter wheat. The correlation analysis results of vegetation index are shown in **Figure 3A**. All vegetation index values exhibit an extremely significant positive correlation with LAI at the p< 0.001 level. The correlation coefficients range from 0.68 to 0.83, with NDRE, LCI, and RVI showing higher correlation coefficients, all surpassing 0.8. The NDVI has the lowest correlation coefficient (0.68), while other vegetation indices range between 0.7 and 0.8. Regarding the correlation among vegetation indices, the correlation coefficient between DVI and NDRE is approximately 0.7, and all other vegetation indices exhibit a correlation coefficient greater than 0.8. Correlation analysis results of texture features are presented in **Figure 3B**. Among all texture features, nir_mean, red_sec, red_cor, and red_hom exhibited positive correlations with LAI, while other texture features showed negative correlations with LAI. The absolute correlation coefficients for all texture features ranged from 0.48 to 0.67. The nir_mean and red_sec had the highest correlation coefficients, both exceeding 0.6, while red_mean had the lowest absolute correlation coefficient (only 0.48). Regarding the correlation among texture features, except for nir_mean, nir_cor, and a few other texture features, the correlation among other texture features is relatively low (most absolute coefficients below 0.5). Overall, the absolute values of the correlation coefficients for vegetation indices are all greater than those for texture features.

### 3.2 Analysis of accuracy of model estimation

**Figure 4:**
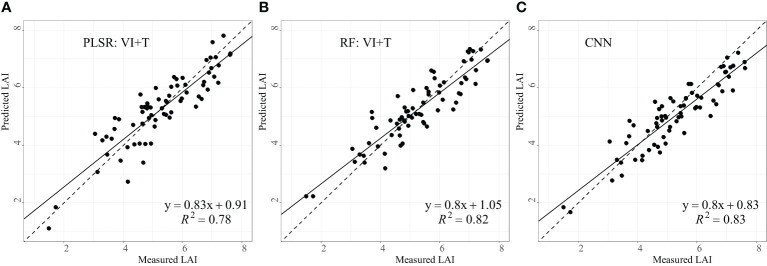
Scatter plots of measured and estimated LAI values of PLSR **(A)**, RF **(B)** and CNN **(C)**. All the statistical analyses in the figure above pass the significance test at the p< 0.001 level.

To assess the impact of different input variable types on the accuracy of winter wheat LAI estimation, three types of combination for input variables were employed in constructing and validating the accuracy of PLSR and RF models. The three input variables were vegetation index (VI), texture feature (T), and the combination of the two variables (VI+T). Both VI and T were the outcomes of the aforementioned feature optimization. The results of model estimation for different input variables are presented in **Table 4**. In the PLSR model, the highest estimation accuracy is achieved when the input variable is VI+T, with *R* ^2^, RMSE, and MAE of 0.78, 0.52, and 0.63, respectively. In the RF model, the highest estimation accuracy is achieved when the input variable is VI+T or VI, both with *R* ^2^ of 0.82. When the input variable is T, the estimation accuracy is the lowest, and *R* ^2^ is only 0.66. On the whole, better LAI inversion results can be obtained by combining VI and T in both PLSR and RF models. As for the CNN model, high estimation accuracy can be achieved based on the scaled raw image data, with the *R* ^2^ reached 0.83. Optimal inversion results were selected for the three models, as shown in **Figure 4**. The fitting regression slopes for three models are all less than and close to 1. This suggests that the three models tend to overestimate in the low value region and underestimate in the high value region. Additionally, the regression lines for PLSR and RF intersect the 1:1 line at a measured LAI of approximately 5, whereas the regression lines for CNN intersect the 1:1 line at a measured LAI of around 4.

**Table 4:** LAI estimation results of winter wheat with different input variables.

Model	Input variable	*R* ^2^	RMSE	MAE
PLSR	VI	0.75	0.54	0.69
	T	0.70	0.59	0.75
	VI+T	0.78	0.52	0.63
RF	VI	0.82	0.47	0.57
	T	0.66	0.61	0.77
	VI+T	0.82	0.46	0.58
CNN	Scaled raw image	0.83	0.50	0.61

All the statistical analyses in the table pass the significance test at the p< 0.001 level.

### 3.3 Spatial distribution of LAI estimation results for winter wheat

**Figure 5:**
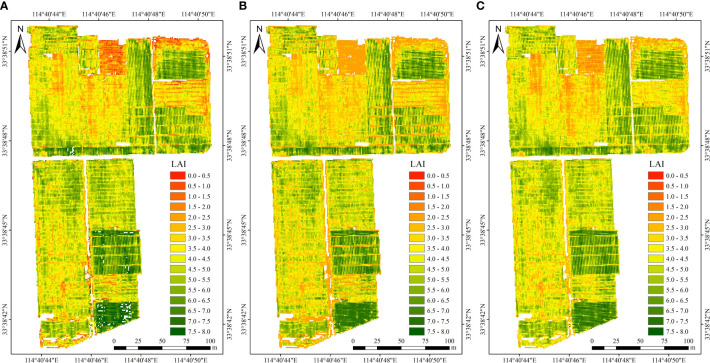
LAI spatial distribution map estimated by PLSR **(A)**, RF **(B)** and CNN **(C)**.

Using the three optimal inversion models, we estimated the spatial distribution of LAI of wheat in the study area, as shown in **Figure 5**. As observed from the result, variations in wheat planting varieties, planting methods, planting density, etc., lead to substantial spatial distribution differences in LAI for wheat growing under the same environmental conditions and management practices. On the whole, the spatial distribution pattern of wheat LAI inversion by the three models was similar, and the high values (larger than 5) were concentrated in the southern and eastern parts of the study area. However, regions with LAI less than 4 were mainly found in the northwestern and eastern parts of the study area. In these regions, PLSR estimation results exhibit lower values compared to the other two models in areas with low LAI values.

### 3.4 Correlation analysis of inversion results of different models

**Figure 6:**
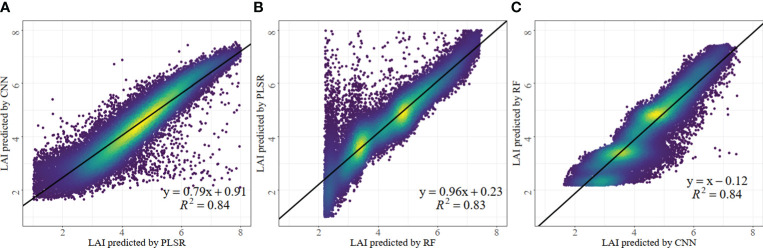
Scatter plot of correlation analysis of LAI estimation results of three models. PLSR **(A)**, RF **(B)** and CNN **(C)**. All the statistical analyses in the figure above pass the significance test at the p< 0.001 level.

Scatter plots illustrating the LAI estimation results for the three models are presented in **Figure 6**. The figure indicates a strong pairwise correlation (*R* ^2^ > 0.8) among the estimated results for the three models. In **Figure 6A**, the correlation between PLSR and CNN is high (*R* ^2^ = 0.84), and the points are evenly distributed near the model fitting line, while the slope only measures 0.79. This suggests that although there is a high correlation between the two models, the similarity in LAI estimates is relatively low. Discrete points at the lower right of the fitting line suggest that PLSR tends to have higher LAI estimates than CNN in specific regions. As shown in **Figure 6B**, the *R* ^2^ of the scatter plot for RF and PLSR estimates is 0.83, but there is an uneven distribution for points. The slope of the fitted line is 0.96, indicating that the estimated values are highly similar for both models. The numerous discrete points in the upper-left imply that PLSR tends to yield higher LAI estimates than RF in specific regions. As shown in **Figure 6C**, the scatter plot *R* ^2^ of the CNN and RF estimation results is 0.84, and the point distribution is also uneven, which is similar to the results of the correlation analysis between RF and PLSR. However, the slope of the fitted line is approximate to 1, which is basically consistent with the 1:1 curve, indicating that the CNN and RF models have the highest similarity in the LAI estimation results. In addition, there are only a few discrete points in the lower right of the fitted line, further indicating that the numerical distributions of the two models are very close to each other.

## 4 Discussion

### 4.1 Comparison of LAI inversion using vegetation index and texture features

To enhance the accuracy of LAI inversion in wheat cultivation, this study employed multispectral images captured by UAV to evaluate the potential of vegetation indices and texture features, and then we evaluated the estimation accuracies of classical machine learning (PLSR, RF) models and deep learning (CNN) model. The results showed that vegetation indices exhibit higher correlation than texture features in LAI estimation. The strong correlation likely stems from vegetation indices being based on the linear combination of spectral reflectance, which partially captures the photosynthetic capacity of vegetation. These findings aligns with studies like Han, et al. (Han et al., 2022), who pointed out that vegetation indices had excellent performance for LAI estimation. However, the results of this study differ from those of earlier studies (Ilniyaz et al., 2023; Fan et al., 2023). These discrepancies may be attributed to the sensitivity of texture features to factors such as vegetation type and image resolution. Moreover, the vegetation indices employed in this study also differ from those used in previous studies, potentially contributing to the observed variations in results. While the LAI estimation accuracy using combined spectral and textural features is higher than that using either features alone, the overall accuracy improvement is not substantial. The marginal enhancement in accuracy observed when combining spectral and textural features may be attributed to the fact that both feature sets are already strongly correlated with LAI. Consequently, their aggregation does not elicit a substantial synergistic effect. The findings of this study are consistent with previous research (Zhang et al., 2022b), which has also demonstrated that combination of spectral and texture features is limited to the improvement of LAI estimation accuracy. Additionally, we also found that among the ten texture features obtained after filtering, eight were derived from the red light band, while only two were derived from the near-infrared band. This finding suggests that the red light band exhibits a stronger correlation with LAI, indicating that the wheat canopy is more sensitive to the red light band.

### 4.2 Performance of the three LAI inversion model

In this study, traditional machine learning and deep learning models were used to estimate wheat LAI. The results demonstrated that the CNN model achieved the highest accuracy, followed by the RF model and the PLSR, which exhibited the lowest accuracy. This outcome aligns with previous studies indicating superior accuracy of the CNN model compared to conventional machine models (Wittstruck et al., 2022; Yamaguchi et al., 2021). In contrast to conventional machine learning approaches, a distinguishing feature of the CNN model is its ability to extract features directly from raw data (Xu et al., 2021), eliminating the need for laborious manual feature selection and cumbersome computation processes. That might be the reason why CNN outperforms other models. Extensive studies have revealed that the relationships between LAI and spectral information are intrinsically nonlinear (Ma et al., 2022; Gao et al., 2023). In general, the RF regression model demonstrates remarkable robustness in handling high-dimensional data and nonlinear relationships, while PLSR, being a linear regression method, is not adequately equipped to capture these intricate nonlinear relationships between spectral reflectance and LAI. As a result, PLSR exhibits inferior accuracy in estimating LAI compared to the RF model.

Despite the high inversion accuracy achieved by the three models in this study, it was observed that at high LAI values (**Figure 4**), the majority of points in the scatter plot deviated below the 1:1 line, indicating a tendency for underestimation by the models. The primary cause of this underestimation lies in the fact that when LAI values are high, the vegetation canopy density typically increases, making it challenging for light to penetrate into the vegetation interior, leading to saturation effects, thus hindering the ability of images to capture wheat canopy information (Wittstruck et al., 2022; Li et al., 2021). On the other hand, the imbalanced distribution of samples with varying LAI values in the dataset may also contribute to the underestimation of LAI values. For instance, the dataset in this study contains fewer samples with high LAI values compared to those with medium and low LAI values, potentially leading to insufficient learning of spectral features associated with high LAI values in the models.

### 4.3 Limitations and future work

Our proposed models demonstrate high precision in estimating the LAI of small-scale crops. Leveraging similar small-scale data, it can promptly offer validation and serve as a reference for the estimation of LAI on a larger scale using satellite remote sensing data, such as Landsat and Sentinel. Despite the promising results obtained in this study, it is important to acknowledge certain limitations. Firstly, the study exclusively utilizes UAV images of winter wheat during a single growth period, restricting the validation and testing of the model’s ability to generalize across diverse growth stages of winter wheat. This is a crucial aspect for the practical application of the model in agricultural management, as it ensures that the model can accurately estimate LAI across the entire growth cycle (Li et al., 2023). Another critical aspect is the absence of comparison with mature pre-trained deep learning models. Pre-trained models are models that have been previously trained on a vast dataset and can be applied to various tasks. This ability to transfer learned features across different problems is a key advantage of deep learning compared to traditional shallow learning methods (Chollet et al., 2022). Thirdly, researchers have pointed out that vegetation height is correlated with aboveground biomass (AGB), and can be used for AGB estimation (Liu et al., 2022b; Li et al., 2020). As AGB is closely related to LAI, vegetation height can also be employed for LAI inversion (Wittstruck et al., 2022). Subsequent research may explore incorporating similar features as model inputs. Finally, it is important to note that the measured values of LAI were obtained indirectly, which introduce some error compared to direct destructive measurements. Therefore, future studies should consider the potential accumulation of errors between these two measurement methods.

## 5 Conclusion

This study compares the performance of classical machine learning (PLSR and RF) methods and deep learning (CNN) approach in estimating the LAI of winter wheat using multispectral images captured by UAV. The results indicate that for PLSR and RF methods, the estimation accuracy is significantly higher when using vegetation indices compared to texture features. The highest accuracy is achieved by combining both vegetation indices and texture features. For the PLSR model, the *R* ^2^ obtained using only vegetation indices or texture features are 0.75 and 0.70, respectively. While for the RF method, the model’s *R* ^2^ for using vegetation indices (0.82) is significantly larger than that using texture features (0.66). Overall, the highest accuracy is achieved by combining both vegetation indices and texture features, with *R* ^2^ values of 0.78 and 0.82 for PLSR and RF, respectively. In contrast to the classical machine learning methods, the CNN method exhibits superior LAI estimation accuracy (*R* ^2 =^ 0.83), demonstrating its ability to effectively extract complex relationships between spectral features and LAI. Moreover, the spatial distribution and numerical values of LAI estimation results from the RF and CNN models exhibit a high degree of similarity, suggesting that both methods capture the spatial patterns of LAI well. In contrast, the PLSR model’s results differ significantly from the other two models. In summary, this study successfully employs CNN in conjunction with multispectral images from UAV to accurately estimate the winter wheat LAI. This approach offers a rapid and cost-effective method for monitoring winter wheat growth, serving as a valuable reference for winter wheat field management.

## Data availability statement

The original contributions presented in the study are included in the article/supplementary material. Further inquiries can be directed to the corresponding author.

## Author contributions

JZ: Conceptualization, Software, Writing – original draft, Writing – review & editing. HY: Conceptualization, Software, Writing – original draft. JW: Writing – original draft. WC: Writing – original draft. YY: Conceptualization, Writing – review & editing.
